# Profiling the unique protective properties of intracranial arterial endothelial cells

**DOI:** 10.1186/s40478-019-0805-4

**Published:** 2019-10-14

**Authors:** Dorien M. A. Hermkens, Olga C. G. Stam, Nienke M. de Wit, Ruud D. Fontijn, Aldo Jongejan, Perry D. Moerland, Claire Mackaaij, Ingeborg S. E. Waas, Mat J. A. P. Daemen, Helga E. de Vries

**Affiliations:** 1 Department of Pathology, Amsterdam UMC, University of Amsterdam, Amsterdam Cardiovascular Sciences, Meibergdreef 9, Amsterdam, Netherlands; 2 Department of Molecular Cell Biology and Immunology, Amsterdam UMC, Vrije Universiteit Amsterdam, Amsterdam Neuroscience, de Boelelaan, 1117 Amsterdam, Netherlands; 3 Department of Clinical Epidemiology, Biostatistics and Bioinformatics; Amsterdam Public Health, Amsterdam UMC, University of Amsterdam, Meibergdreef 9, Amsterdam, Netherlands

**Keywords:** Vascular cognitive impairment, Intracranial vasculature, Endothelial cells, RNA sequencing

## Abstract

Cardiovascular disorders, like atherosclerosis and hypertension, are increasingly known to be associated with vascular cognitive impairment (VCI). In particular, intracranial atherosclerosis is one of the main causes of VCI, although plaque development occurs later in time and is structurally different compared to atherosclerosis in extracranial arteries. Recent data suggest that endothelial cells (ECs) that line the intracranial arteries may exert anti-atherosclerotic effects due to yet unidentified pathways. To gain insights into underlying mechanisms, we isolated post-mortem endothelial cells from both the intracranial basilar artery (BA) and the extracranial common carotid artery (CCA) from the same individual (total of 15 individuals) with laser capture microdissection. RNA sequencing revealed a distinct molecular signature of the two endothelial cell populations of which the most prominent ones were validated by means of qPCR. Our data reveal for the first time that intracranial artery ECs exert an immune quiescent phenotype. Secondly, genes known to be involved in the response of ECs to damage (inflammation, differentiation, adhesion, proliferation, permeability and oxidative stress) are differentially expressed in intracranial ECs compared to extracranial ECs. Finally, Desmoplakin (DSP) and Hop Homeobox (HOPX), two genes expressed at a higher level in intracranial ECs, and Sodium Voltage-Gated Channel Beta Subunit 3 (SCN3B), a gene expressed at a lower level in intracranial ECs compared to extracranial ECs, were shown to be responsive to shear stress and/or hypoxia. With our data we present a set of intracranial-specific endothelial genes that may contribute to its protective phenotype, thereby supporting proper perfusion and consequently may preserve cognitive function. Deciphering the molecular regulation of the vascular bed in the brain may lead to the identification of novel potential intervention strategies to halt vascular associated disorders, such as atherosclerosis and vascular cognitive dysfunction.

## Introduction

Cardiovascular diseases (CVD) such as atherosclerosis, hypertension and heart failure are all associated with an increased risk of vascular cognitive impairment (VCI). CVD can influence cerebral perfusion and may also lead to development of white matter lesions, blood-brain-barrier dysfunction, cerebral microbleeds, brain atrophy and neuro-inflammation [16, 17, 65]. Interestingly, improvement of heart function or lowering of blood pressure is known to stimulate cognitive functioning [11, 23, 42]. Therefore, the key to successful therapeutic intervention in VCI requires detailed understanding of the underlying cardiovascular mechanisms.

One of the main causes of VCI is atherosclerotic plaque formation in the vessel wall [4, 5, 36, 63]. Atherosclerosis is the most well-known disease of the arteries and is known to manifest itself differently in intracranial arteries compared to extracranial arteries. In humans, intracranial atherosclerosis develops approximately 20 years later than extracranial atherosclerosis [57]. Furthermore, intracranial atherosclerotic plaques are less advanced and less vulnerable to plaque rupture and intraplaque hemorrhages, compared to extracranial atherosclerotic plaques [31, 33, 44], suggesting a reduced susceptibility of intracranial arteries to systemic risk factors for atherosclerosis. However, current research on VCI is focused on the vasculature in general (recently reviewed in [14, 26]) and does not describe the specific role of the intracranial arteries.

Endothelial cells (ECs) form a single cell layer that lines all blood vessels and regulates exchange between the bloodstream and surrounding tissues. Intracranial ECs display an increased antioxidant enzyme activity and a strong blood-brain-barrier that protect the brain vasculature and the underlying brain parenchyma from disease development [9]. To unravel the role of intracranial ECs in cerebral vascular functioning on a molecular level, several studies have analyzed the transcriptome of brain microvascular ECs, mostly in mice [20, 24, 28, 32, 48, 60]. So far, only a limited number of studies describing the transcriptome of human astrocytes, microglia, neurons, oligodendrocytes, pericytes and endothelial cells has been performed [8, 52, 56, 66]. To date, all studies on human brain samples are focused on the microvasculature of the brain. Although the larger intracranial arteries are key in atherosclerotic plaque formation and consequently VCI development, data on the transcriptome of the ECs of the intracranial arteries are lacking. Uncovering the endothelial mechanisms that contribute to intrinsic atheroprotective properties and thus to lowering of the local intracranial atherosclerotic plaque burden, might be key to healthy cerebral vascular functioning.

With our study we are the first to unravel the distinct molecular pathways of intracranial artery endothelial cells in humans in order to identify novel pathways possibly involved in the protective nature of these ECs in the development of atherosclerosis and VCI. The paired comparison of the ECs derived from the intracranial artery as well as from the extracranial artery has to our knowledge not been performed before and will be addressed here. The identification of protective pathways in the vascular bed of the brain is a first step towards novel early intervention strategies to counteract brain endothelial associated diseases like atherosclerosis and VCI.

## Materials & methods

### Patient material

We obtained post-mortem samples from the common carotid artery (CCA) and the basilar artery (BA) from 15 individuals, who were autopsied at the Amsterdam UMC in the period between February 2015 and July 2016. Inclusion criteria were 1) all individuals were adults, 2) time between death and autopsy (post-mortem delay, PMD) was less than 72 h and 3) written permission to obtain the materials at autopsy for research purposes was granted by the family of the patients. Age, gender, cause of death and PMD were documented in Table 1. The criteria for the code of proper secondary use of human tissue in the Netherlands were met *[Human tissue and medical research: code of conduct for responsible use. Federation of Dutch Medical Scientific Societies].* During autopsy the circle of Willis and the carotids were exposed and macroscopically normal parts of the CCA and BA were sampled. The materials were stored in containers and frozen immediately using liquid nitrogen. After autopsy the samples were stored in a freezer at − 80 °C.

**Table 1:** Patient characteristics with patient age, sex, cause of death and post-mortem delay. Patient 1–9 were used for RNA sequencing, patient 10–15 for biological validation. The cause of death of 4 patients is unknown, since the arteries for the study were donated anonymously and only age, gender and post mortem delay were available

Patient number	Age (years)	Sex (male/female)	Cause of death	Post mortem delay (hours)
1	60	m	ALS	< 24
2	59	m	ALS	< 12
3	78	m	unknown	< 12
4	87	f	Stroke	< 6
5	65	f	ALS	24–48
6	62	f	ALS	24–48
7	66	f	ALS	24–48
8	32	m	unknown	24–48
9	68	m	ALS	< 24
10	69	m	Sepsis	< 24
11	68	f	Pneumonia	24–48
12	46	f	Peritonitis	48–72
13	85	f	Stroke	48–72
14	88	f	Unknown	48–72
15	59	m	Unknown	< 24

### Cryosectioning and hematoxylin staining

The artery specimen were embedded with Tissue Tek (Tissue-Tek O.C.T Compound, Sakura) and liquid nitrogen on cryomolds. The membrane slides (MembraneSlide 1.0 PEN, Carl Zeiss Microscopy GmbH) were pretreated with UV light for 15 min. Frozen sections of 8 μm were cut and mounted on the slides. The slides were stored at − 80 °C. To enhance the visibility of the EC, the frozen sections were stained with hematoxylin (Mayer’s hematoxylin Solution, Sigma Aldrich). To prevent the slides from defrosting, the hematoxylin, ethanol solutions and xylene were precooled. The slides were stained and dehydrated with graded ethanol solutions (75, 95 and 100%) ending with xylene. Afterwards the slides were allowed to dry for a maximum of 5 min in the hood. The stained slides were stored at − 80 °C.

### Laser capture microdissection

Laser capture microdissection (LCM6, Leica) was used to isolate the endothelial cells. At a 100x magnification the arteries were scanned for areas were the endothelial lining was intact and at 200x magnification the areas were selected for laser dissection. Areas which showed inflammation or accumulation of macrophages were excluded. In order to prevent the laser beam from damaging the EC layer, the cut was made directly underlying the luminal ECs. Damaged endothelium, defined as a non-continuous endothelial layer, was excluded. The samples were collected in extraction buffer (PicoPure RNA isolation kit (ThermoFisher) in a RNA-free microcentrifuge tube.

### RNA isolation and cDNA amplification

RNA isolation was performed using the PicoPure RNA isolation kit (ThermoFisher) using the manufacturers’ instructions. After incubating for 30 min at 42 °C, the sample was centrifuged at 800 x g for 2 min and stored at − 80 °C. Ethanol (70%) was added in a 1:1 ratio to the cell extract. For optimal precipitation, samples were stored for 30 min. at − 20 °C before adding to the purification column. DNAse treatment was performed on the purification column. RNA was eluted in a volume of 11 μl. For cDNA synthesis we used the Ovation® RNA-Seq System V2 (Nugen) using the manufacturers’ instructions. Purification of SPIA cDNA was performed with the QIAquick PCR Purification Kit (Qiagen).

### RNA sequencing

RNA sequencing was performed by Service XS (GenomeScan) using the Illumina Next Generation Sequencing Technology. The NEBNext® Ultra II DNA Library Prep kit for Illumina (cat# NEB #E7645S/L) was used to process the samples. Fragmentation of the DNA using the Biorupor Pico (Diagenode), ligation of sequencing adapters, and PCR amplification of the resulting product was performed according to the procedure described in the NEBNext Ultra DNA Library Prep kit for Illumina Instruction Manual. The quality and yield after sample preparation was measured with the Fragment Analyzer. The size of the resulting product was consistent with the expected size of approximately 500–700 base pairs. Clustering and DNA sequencing using the Illumina NextSeq500 was performed according to manufacturer’s protocols. A concentration of 1.6 pM of library was used. NextSeq control software 2.2.0 was used. Image analysis, base calling, and quality check was performed with the Illumina data analysis pipeline RTA v2.4.11 and Bcl2fastq v2.20.

### RNA sequencing data analysis

A dataset with a mean of 1.9 Gb (25.8 million reads) of Illumina-filtered single end 75 base pairs sequence data was generated per sample. Data analysis was performed using R (v3.3.1) and Bioconductor (v3.4). Raw sequencing data were subjected to quality control using FastQC (https://www.bioinformatics.babraham.ac.uk/projects/fastqc/), dupRadar [50], Picard Tools (http://broadinstitute.github.io/picard/) and trimmed using Trimmomatic (v0.32; http://www.usadellab.org/cms/?page=trimmomatic). Reads were aligned to the human reference genome hg38 using HISAT2 (v2.0.4; https://ccb.jhu.edu/software/hisat2/index.shtml).

After alignment, samples of two individuals were excluded due to low read coverage. Possible mRNA degradation of the remaining samples was assessed by quantifying the 3′ bias of each gene using mRIN [12]. The summary mRIN values of the samples from the remaining 9 individuals showed no significant RNA degradation (*P*-value > 0.05) (Additional file 1: Table S1) and no correlation between PMD and mRIN values (Additional file 1: Figure S3).

Gene level counts were obtained using HTSeq (v0.6.1; https://github.com/simon-anders/htseq) and the human GTF from Ensembl (release 85; excluding mitochondrial-encoded genes). Statistical analyses were performed using the edgeR and limma R/Bioconductor packages [43, 45]. Genes with more than 5 counts in 6 or more samples were retained. Count data were transformed to log2-counts per million (logCPM), normalized by applying the trimmed mean of M-values method and precision weighted using voom [30]. Differential expression was assessed using an empirical Bayes moderated t-test within limma’s linear model framework including individual as a blocking variable. Resulting *P*-values were corrected for multiple testing using the Benjamini-Hochberg false discovery rate (FDR). Additional gene annotation was retrieved from Ensembl (release 89) using the biomaRt R/Bioconductor package. Raw sequence data will be available in the European Genome-Phenome Archive (EGA) and upon request.

Gene set enrichment analysis was performed using CAMERA (limma package) with preset value of 0.01 for the inter-gene correlation using gene set collections Hallmark, C2 (curated), C3 (motifs), C5 (gene ontology), C6 (oncogenic) and C7 (immunologic) retrieved from the Molecular Signatures Database (MSigDB v6.0; Entrez Gene ID version) [55, 64]. Resulting *P*-values were corrected for multiple testing using the Benjamini-Hochberg FDR. For functional annotation of genes related to EC damage the following gene ontology (GO) terms were used: regulation_of_vascular_permeability (go:0043114), response_to_oxidative_stress (go:0006979), inflammatory_response (go:0006954), endothelial_cell_differentiation (go:0045446), regulation_of_endothelial_cell_proliferation (go:0001936), regulation_of_cell_adhesion (go:0030155), regulation_of_cellular_response_to_hypoxia (go:1900037). For functional annotation of genes related to perfusion the following terms were used: regulation_of_blood_pressure (go:0008217), regulation_of_blood_circulation (go:1903522), mechanosensory_behavior (go:0007638), mechanoreceptor_differentiation (go:0042490), response_to_fluid_shear_stress (go:0034405). For functional annotation of genes related to cognition the following terms were used: cognition (go:0050890), alzheimers_disease (hsa05010, gse1297), aging_brain (gse1572, gse1572).

### RT-qPCR

For validation of the human samples, qPCR was performed on a BioRad CFX384 machine with the ssoFast EvaGreen method (Biorad). Expression levels of transcripts were obtained with LinRegPCR [41, 46]. Expression levels were normalized against CD31 and vWF expression levels, as these genes are the most stable genes between BA and CCA samples, analyzed with geNorm module of qbase+ [22, 47] (Additional file 1: Figure S1). qPCR on cell culture samples was performed on a BioRad CFX384 machine with the SensiFAST SYBR® Green method (Bioline). Expression levels of transcripts were obtained with LinRegPCR [41, 46]. For the shear stress analysis the transcript levels were normalized against Rplp0 and β-actin and for the normoxia-hypoxia analysis against B2M [35]. All primer sequences are listed in Additional file 1: Table S2.

### In vitro cell culture

The human brain endothelial cell line hCMEC/D3 was kindly provided by Prof. dr. Couraud (Institute Cochin, University Paris Descartes, Paris, France). Cells were grown in EGM-2 medium (Lonza, Basel, Switzerland). Cells were subjected to unidirectional laminar shear stress essentially as described [10], with the following modifications. Cells were cultured on collagen type 1- coated parallel plate flow chambers (μ-Slide I 0.4 luer, Ibidi, Martinsried, Germany) and exposed to calibrated shear stress levels of 10 dyne/cm^2^ and 0.74 dyne/cm^2^ during 4 days using the Ibidi pump system (Ibidi, Martinsried, Germany). Culturing of cells under hypoxic conditions at 1% oxygen was performed as described previously [34].

### Immunocytochemistry

For immunocytochemical analysis of the human brain endothelial cell culture, cells were fixated with 4% paraformaldehyde, permeabilized with 0,1% Triton in PBS for 10 min and non-specific binding was blocked with 10% normal goat serum (X0907, DAKO, Santa Clara, CA, USA) for 30 min. Subsequently cells were incubated overnight at 4 °C with primary antibodies against CD31 (DAKO, M0823) and HOPX (Invitrogen, PA-72855), SCN3B (Sigma, HPA04707) or DSP (Sdix, 2282.00.02). Secondary antibodies against goat-anti-rabbit Alexa633 (Invitrogen, A-21070) and goat-anti-mouse IgG1 Alexa568 (Invitrogen, A-21124) together with Hoechst for nuclei staining were incubated for 1 h at room temperature. The representative images were taking using a Leica TCS SP8 X microscope (63x objective, Leica Microsystems, Wetzlar, Germany).

### Immunohistochemistry

For immunohistochemical analysis of the human brain microvasculature, 5 μm cryosections mounted on coated glass slides (Menzel Gläser Superfrost PLUS, Thermo Scientific, Braunschweig Germany), were air-dried and fixated in acetone for 10 min. Sections were incubated for 30 min with 10% normal goat serum. Subsequently, sections were incubated overnight at 4 °C with primary antibodies against HOPX (Invitrogen, PA-72855), SCN3B (Sigma, HPA04707) or DSP (Sdix, 2282.00.02). Subsequently, the secondary antibody goat anti-rabbit Alexa 488 (Life Technologies) was incubated for 1 h. ULEX (Vector Labs) was used as an endothelial cell marker and detected using Alexa 555 labeled streptavidin (Life Technologies). Finally, sections were stained with Hoechst (Molecular Probes) to visualize cellular nuclei and mounted with Mowiol mounting medium. The representative images were taken using a Leica DM6000 microscope (40x objective, Leica Microsystems).

For immunohistochemical analysis of the human BA, 5 μm formalin fixed paraffin tissue was mounted on coated glass slides and dewaxed with xylene and rehydrated trough graded ethanol solutions to water. Antigen retrieval was performed at 98 °C for 20 min in a Lab Vision™ PT-module (ThermoFisher) with Tris-EDTA (TA-250-PM4x, ThermoFisher; pH = 9). The tissue was blocked with Super Block (AAA999, Scytech, Logan, UT, USA) for 10 min at room temperature. Where after the tissues were incubated with primary antibodies against CD31 (DAKO, M0823) and HOPX (Invitrogen, PA-72855), SCN3B (Sigma, HPA04707) or DSP (Sdix, 2282.00.02) overnight at 4 °C. Secondary antibodies goat-anti-rabbit Alexa633 (Invitrogen, A-21070) and goat-anti-mouse IgG1 Alexa568 (Invitrogen, A-21124) together with Hoechst were applied for 30 min at room temperature. Sections were mounted with Prolong gold (P36935, ThermoFisher) and representative images were takes using a Leica TCS SP8 X microscope (40x objective, Leica Microsystems, Wetzlar, Germany).

## Results

### Laser capture microdissection of human intra- vs. extracranial arterial ECs

**Fig. 1:**
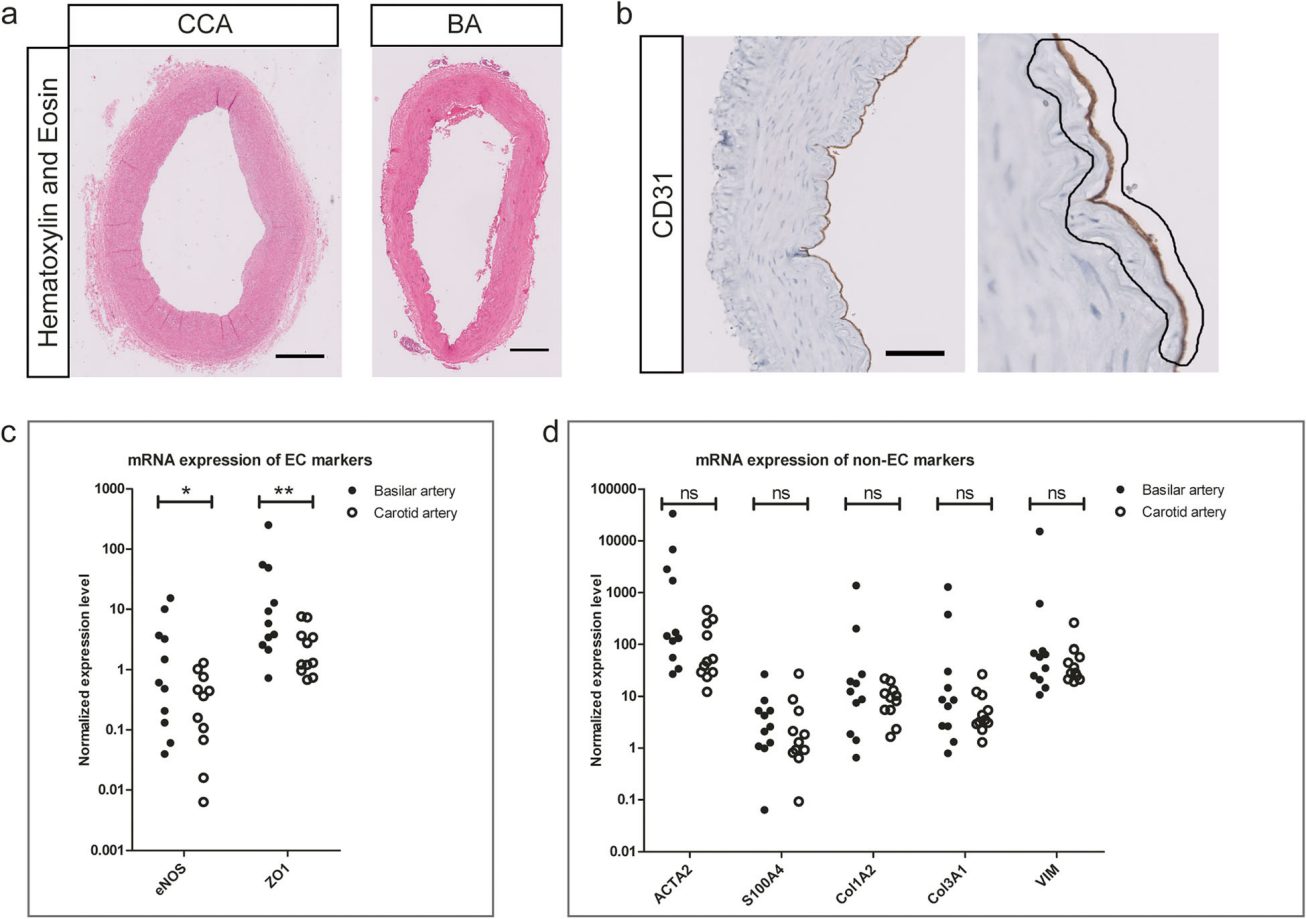
Laser capture microdissection of macroscopically normal CCA and BA (**a**) Cross-sections of macroscopically normal CCA and BA, depicted with Hematoxylin and Eosin staining. Scale bar for CCA and BA represents resp. 1200 and 300 μm. **b** Vessel wall of BA (left, marked by CD31 in brown, scale bar represents 120 μm) and higher magnification vessel wall (right) with selected area depicting representative LCM cutting margins for EC collection. **c** Significant differential expression of eNOS and ZO1 between the BA and CCA measured by qPCR. **d** No significant difference of smooth muscle cell (ACTA2) and fibroblast (S100A4, Col1A2, Col3A1, VIM) marker expression in LCM-collected population of the BA compared to the CCA samples. **c**, **d** Data are depicted as expression level normalized against CD31 and vWF. Significance level determined by Wilcoxon signed rank test, *p* < 0.05 (*) and *p* < 0.01 (**) considered significant. *n* = 11 patients. ACTA2 = Actin alpha 2, CD31 = Cluster of differentiation 31, Col1A2 = Collagen type I alpha 2, Col3A1 = Collagen type Ill alpha 1, eNOS = endothelial Nitric Oxide Synthase, S100A4 = S100 Calcium Binding Protein A4/Fibroblast specific protein 1, VIM = Vimentin, ZO1 = Zona Occludens Protein 1

Analyses were performed on paired post-mortem derived ECs of the intracranial basilar artery (BA) and the extracranial common carotid artery (CCA). The post-mortem samples were derived from 15 individuals with distinct causes of death and age ranging from 32 years to 88 years (Table 1). To specifically detect intrinsic differences in gene expression between intra- and extracranial ECs, rather than disease associated characteristics, analyses were performed by comparing macroscopically normal tissues of intra- and extracranial origin within each individual (Fig. 1a). Laser microdissection was used to isolate the ECs (Fig. 1b).

In order to validate the selection of ECs and the differential expression between the BA and the CCA, we performed qPCR analysis of genes known to be highly expressed in intracranial ECs, such as Zona Occludens 1 (ZO1) and Endothelial Nitric Oxide Synthase 3 (eNOS) [6, 19]. We found a higher expression of ZO1 and eNOS in the intracranial artery ECs compared to extracranial artery ECs (Fig. 1c), whereas Platelet and Endothelial Cell Adhesion Molecule 1 (PECAM1/CD31) and von Willebrand factor (vWF) were equally expressed between the two locations (and used as reference genes, Additional file 1: Figure S1).

By selecting the ECs with laser microdissection, our samples could contain a subset of non-endothelial transcripts. To rule out differences in the non-endothelial subpopulation between the BA and the CCA samples, we performed qPCR for several non-endothelial markers. Gene expression of smooth muscle cells and fibroblast components was not significantly different between the BA and CCA (Fig. 1d).

### An immunoquiescent- and damage protective phenotype of the intracranial artery ECs

**Fig. 2:**
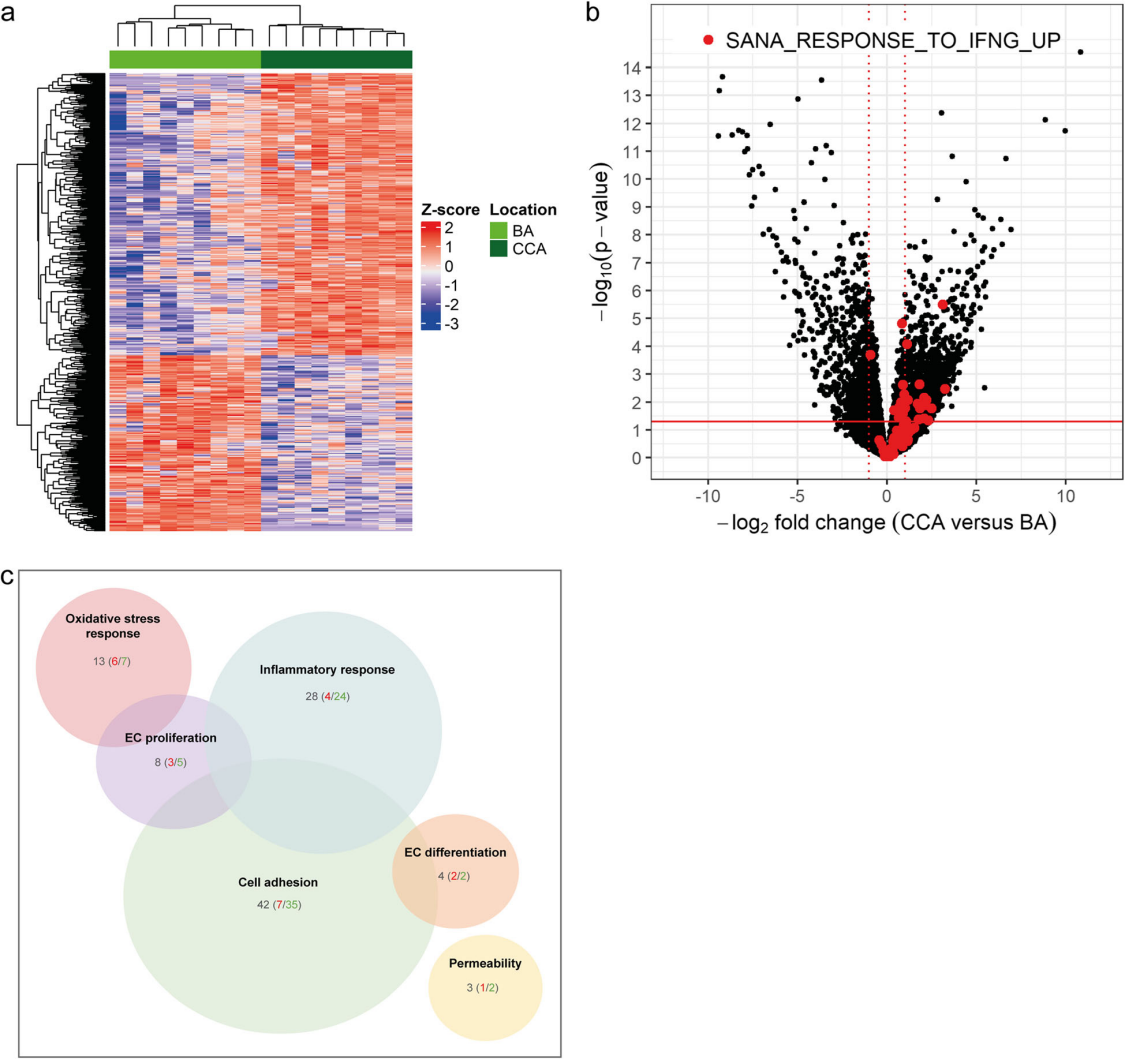
Differential regulation of immune- and EC damage response genes in the ECs of the BA compared to the CCA (**a**) Heatmap of the 593 differentially expressed genes (absolute log2-fold-change > 2 and adjusted *P*-value < 0.05). Dendrogram using Euclidean distance and complete linkage. Color coding corresponds to z-score of logCPM values from blue (downregulated) to red (upregulated). **b** Volcano plot with black dots depicting individual genes. In red, genes that were also found to be upregulated by IFNγ in a published dataset (MSigDB ID: M4551) [49]. Solid red line corresponds to a P-value = 0.05. Dotted red lines correspond to an absolute log2-fold-change of 1. **c** Venn diagram with genes related to EC damage response with absolute log2-fold-change > 2 and *p*-value < 0.05. Numbers in grey are total genes in subgroup, in red are the genes higher expressed in the BA, in green the genes higher expressed in the CCA

We performed RNA sequencing on the ECs of the BA and CCA of nine individuals. Despite the post-mortem nature, the samples of these nine patients did not show significant RNA degradation (*P*-value > 0.05) (Additional file 1: Table S1). Differential gene expression revealed that 900 genes were differentially expressed with absolute log2 fold-change > 2 and P-value < 0.05, and 593 genes were differentially expressed with absolute log2 fold-change > 2 and adjusted *P*-value < 0.05 (Fig. 2a). Gene set enrichment analysis on GO molecular function terms showed that proteins encoded by the genes that were expressed to a higher level in the BA ECs are mainly involved in ribosome functioning and translation. In addition, genes higher expressed in the CCA ECs are mainly involved in antigen binding and receptor activity. Furthermore enrichment analysis on GO cellular component terms confirmed enrichment of ribosomal transcripts in the BA, whereas the genes enriched in the CCA are involved in inflammation (Table 2). This analysis indicates that ECs from intracranial and extracranial arteries differ in their molecular functions.

**Table 2:** Enrichment of molecular function and cellular component GO-terms in BA and CCA ECs. Top 5 GO molecular function and cellular component terms higher expressed in the BA compared to the CCA and vice versa. FDR is false discovery rate.0020

Gene set enrichment analysis on GO molecular function terms			
Top 5 GO-terms higher expressed in basilar artery compared to carotid artery			
G0-term	Molecular function	*P*-value	FDR
GO:0003735	STRUCTURAL_CONSTITUENT_OF_RIBOSOME	6.27E-07	3.51E-04
GO:0003743	TRANSLATION_INITIATION_FACTOR_ACTIVITY	9.51E-06	2.31E-03
GO:0044822	POLY_A_RNA_BINDING	7.67E-05	9.26E-03
GO:0016675	OXIDOREDUCTASE_ACTIVITY_ACTING_ON_A_HEME_GROUP_OF_DONORS	2.00E-04	1.65E-02
GO:0045182	TRANSLATION_REGULATOR_ACTIVITY	2.11E-04	1.67E-02
Top 5 GO-terms higher expressed in carotid artery compared to basilar artery			
G0-term	Molecular function	*P*-value	FDR
GO:0042605	PEPTIDE_ANTIGEN_BINDING	6.42E-05	8.68E-03
GO:0008329	SIGNALING_PATTERN_RECOGNITION_RECEPTOR_ACTIVITY	4.08E-04	2.55E-02
GO:0004896	CYTOKINE_RECEPTOR_ACTIVITY	8.94E-04	3.92E-02
GO:0001653	PEPTIDE_RECEPTOR_ACTIVITY	1.24E-03	4.74E-02
GO:0004115	3_5_CYCLIC_AMP_PHOSPHODIESTERASE_ACTIVITY	1.40E-03	5.10E-02
Gene set enrichment analysis on GO cellular component terms			
Top 5 GO-terms higher expressed in basilar artery compared to carotid artery			
G0-term	Cellular component	*P*-value	FDR
GO:0044391	RIBOSOMAL_SUBUNIT	8.67E-08	1.02E-04
GO:0022626	CYTOSOLIC_RIBOSOME	9.69E-08	1.02E-04
GO:0015934	LARGE_RIBOSOMAL_SUBUNIT	1.65E-07	1.39E-04
GO:0005840	RIBOSOME	2.92E-07	2.02E-04
GO:0015934	CYTOSOLIC_LARGE_RIBOSOMAL_SUBUNIT	2.99E-07	2.02E-04
Top 5 GO-terms higher expressed in carotid artery compared to basilar artery			
G0-term	Cellular component	*P*-value	FDR
GO:0042611	MHC_PROTEIN_COMPLEX	1.90E-06	7.49E-04
GO:0042613	MHC_CLASS_II_PROTEIN_COMPLEX	5.47E-05	8.05E-03
GO:0032281	AMPA_GLUTAMATE_RECEPTOR_COMPLEX	2.00E-04	1.65E-02
GO:0061702	INFLAMMASOME_COMPLEX	3.85E-04	2.49E-02
GO:0005858	AXONEMAL_DYNEIN_COMPLEX	4.33E-04	2.63E-02

Gene set enrichment analysis also identified a top-ranked gene set consisting of genes upregulated in primary endothelial cell types (lung, aortic, iliac, dermal, and colon) treated with Interferon-gamma (IFNγ) (MSigDB ID: M4551) [49]. Almost all genes upregulated by IFNγ were higher expressed in the extracranial ECs compared to the intracranial ECs (Fig. 2b and Additional file 1: Table S3A), which suggests specific regulation of the intracranial ECs resulting in an anti-inflammatory phenotype. This fits with earlier in vitro data showing an immunoquiescent state of brain microvascular ECs [59], supporting the hypothesis that intracranial ECs have a more protective phenotype to resist damage.

To further unravel the molecular pathways involved in the protective nature of the intracranial ECs, we focused on genes involved in EC damage. For this, we analyzed the 900 differentially expressed genes with absolute log2-fold-change > 2 and *p*-value < 0.05 (corresponding to adjusted p-value < 0.236) for their involvement in the following EC damage responses: inflammation, permeability, response to oxidative stress or hypoxia, proliferation, differentiation and cell adhesion (for GO-terms see Materials and Methods section). For almost all of these processes, except for hypoxia responses, several genes were differentially expressed (Fig. 2c and Additional file 1: Table S3B): 28 genes involved in inflammation, 42 genes in cell adhesion, 3 genes in permeability, 13 genes in oxidative stress, 8 genes in EC proliferation, 4 genes in EC differentiation, suggesting a different response to EC damage in intra- and extracranial ECs. For the inflammatory genes, 24 of the 28 genes did show lower expression levels in the intracranial ECs, as did 35 of the 42 genes involved in cell adhesion, which again fits with the protected state of the intracranial ECs mentioned before. Thus, analysis of our dataset suggests that intracranial artery ECs display protective mechanisms by which an immunoquiescent phenotype is established. In addition, the data show that EC differentiation, permeability and the response to oxidative stress are differentially regulated in intracranial versus extracranial ECs.

### Perfusion and cognition-related gene expression in the intracranial artery ECs

**Fig. 3:**
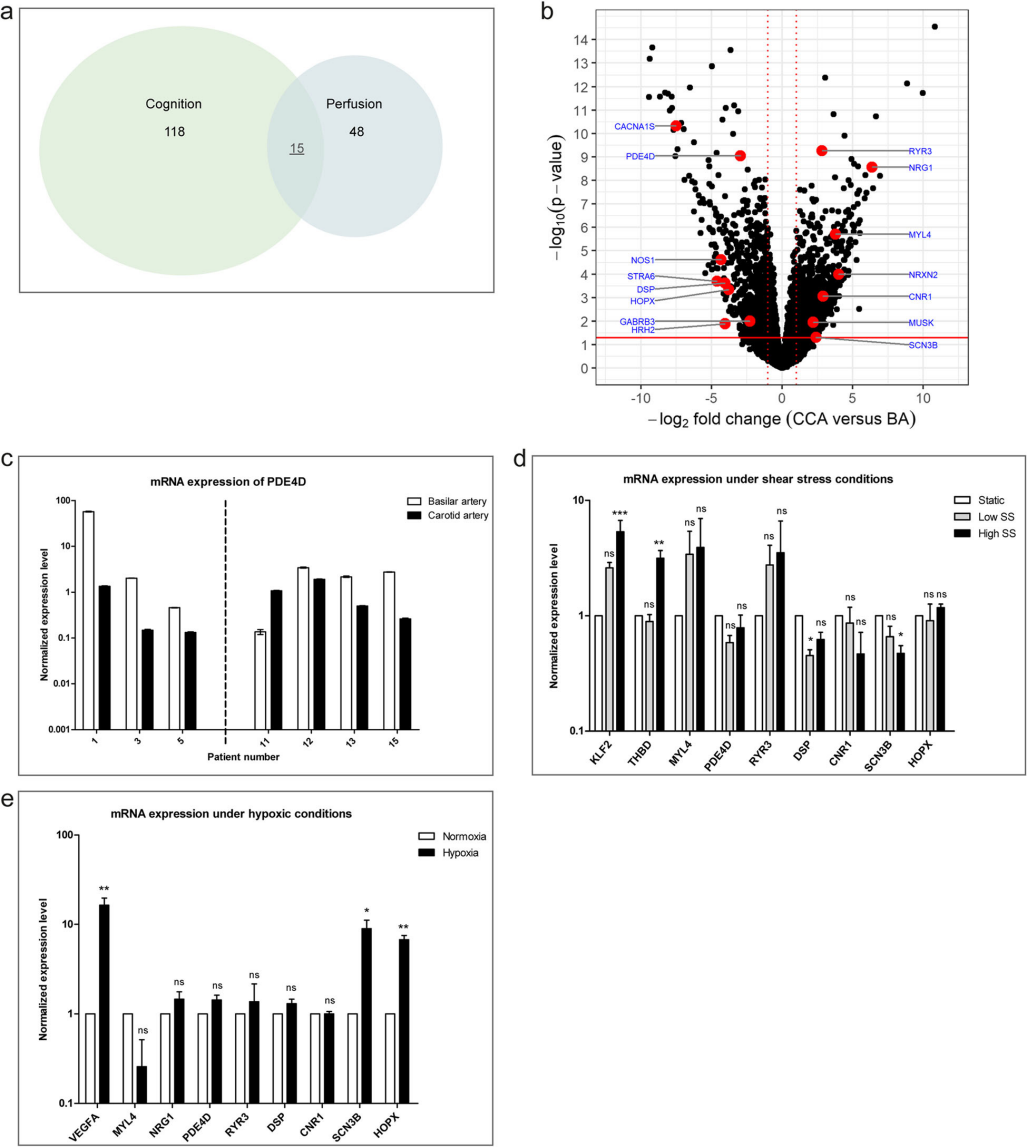
A role for DSP, HOPX and SCN3B in EC shear stress and/or hypoxia regulation (**a**) Venn diagram of differentially expressed genes that are involved in cognition (118 genes), perfusion (48 genes) or both (15 genes, underscored) with absolute log2-fold-change > 2 and p-value < 0.05. **b** Volcano plot of RNA sequencing data with black dots depicting individual genes. In red the genes differentially expressed and involved in perfusion and cognition. Solid red line corresponds to a P-value = 0.05. Dotted red lines correspond to an absolute log2-fold-change of 1. **c** Technical and biological validation with qPCR of one representative gene (*n* = 3 technical replicates). Patients 1, 3, 5 were used in RNA sequencing experiment (technical validation), patient 11, 12, 13, 15 were not used in RNA sequencing experiment (biological validation). Expression is normalized against CD31 and vWF expression. Dashed line indicates two independent experiments. **d** The effect of shear stress conditions on gene expression. KLF2 and THBD are used as positive controls. SCN3B and DSP are significantly downregulated upon shear stress conditions (*n* = 3–5 shear stress experiments, Friedman test). static = 0 dyne/cm2, low = 0.75dyne/cm2, high = 10dyne/cm2. Expression of individual genes is normalized against Rplp0 and B-actin expression and compared to the static condition. **e** The effect of hypoxic conditions on gene expression. VEGFA is used as positive control. SCN3B and HOPX are significantly upregulated upon hypoxia (*n* = 5 hypoxia experiments, Paired t-test). Normoxia = 20% O2, hypoxia = 1% O2. Expression of individual genes is normalized against B2M expression and compared to the normoxia condition. **c**, **d** and **e** Mean with SEM is depicted. **d**, **e** Significance level of *p* < 0.05 (*), *p* < 0.01 (**) and *p* < 0.001 (***) is considered significant. For full genes names see Additional file 1: Table S2

Cerebral circulatory dysfunction is the common nominator for VCI and atherosclerosis. Since the intracranial ECs display a damage protective phenotype, we here investigated whether the intracranial ECs can also exert their protective function upon circulatory dysfunction. For this, we selected a subset of 48 genes matching the GO-terms ‘blood pressure, blood circulation, mechanosensing and shear stress’ (see Materials and Methods) from the 900 differentially expressed genes with absolute log2-fold-change > 2 and *p*-value < 0.05 (corresponding to adjusted p-value < 0.236). Since we were also interested in the involvement of circulatory dysfunction in cerebral function, we additionally selected for the involvement of these genes in ‘cognition, Alzheimer’s, aging brain’ using the homonymous search terms (see Materials and Methods, 118 genes), resulting in a set of 15 genes (Fig. 3a and Additional file 1: Table S3C). Of these 15 genes, eight genes were expressed at a higher level in the BA compared to the CCA and seven genes were expressed at a higher level in the CCA compared to the BA (Fig. 3b). Of these 15 genes, only three (NRG1, NOS1, PDE4D) have already been described for their involvement in EC damage.

To validate the differential expression between intra- and extracranial ECs of these 15 genes known to be involved in perfusion and cognition, we performed quantitative PCR for both technical validation (in three individuals from our RNA sequencing analysis) and biological validation (four individuals not included in our RNA sequencing analysis) (Table 1 for patient characteristics). We could technically and biologically validate the differential expression of five genes (CNR1, GABRB3, PDE4D, RYR3 and STRA6) with qPCR in the majority of individuals (Fig. 3c and data not shown). The expression of the other genes could not be validated in the human arteries with qPCR due to levels below the qPCR threshold or due to technical reasons.

### A novel role for DSP, HOPX and SCN3B in EC shear stress and/or hypoxia responsiveness

Subsequently we studied the susceptibility of the 15 genes to perfusion in vitro by analyzing their mRNA expression under shear stress and hypoxic conditions. Due to technical reasons or low expression levels of these genes in the in vitro brain ECs, out of the 15 genes, eight genes could be functionally studied further for their susceptibility to hypoxia and seven genes for shear stress (Fig. 3d, e). For shear stress conditions, intracranial ECs were grown under static conditions and under low- and normal shear stress (0.75 dynes/cm^2^ and 10 dynes/cm^2^, respectively) [3]. Shear stress is known to induce an atheroprotective phenotype in ECs [7, 40]. As shear stress increased, the expression of the genes of interest changed; the extracranial more highly expressed gene SCN3B and the intracranial more highly expressed gene DSP were significantly downregulated upon resp. high and low shear stress (*n* = 5) (Fig. 3d). SCN3B inactivates sodium channels and thereby regulates the action potentials in neurons and myocytes. Low expression of SCN3B is observed in non-excitable cells, whereas expression in ECs was only detected once before in arterial mouse intracranial ECs, however its physiological role in these non-excitable cells is unclear [18, 54, 60]. Our data suggest a role for SCN3B in human intracranial ECs and elucidate its susceptibility to shear stress. Whereas DSP is known to be involved in adhesion, the function of DSP has not been investigated before in intracranial ECs. Our data suggests that DSP is susceptible to shear stress in intracranial ECs.

We furthermore tested the expression of these genes under hypoxic conditions (1% O2) and control conditions (20% O2) (Fig. 3e, *n* = 3–5) [35]. HOPX and SCN3B revealed an increased expression under hypoxic conditions. This showed the responsiveness of SCN3B, not only for shear stress, but also for hypoxia. HOPX was only once detected in the ECs of the cerebral cortex [53], however no physiological role of HOPX in ECs is known. We here revealed the responsiveness of HOPX for hypoxic conditions. With our in vitro data we could demonstrate that SCN3B, DSP and HOPX are responsive to shear stress and/or hypoxia in intracranial ECs, besides being differently regulated in the intracranial ECs compared to the extracranial ECs. These genes were selected for their involvement in cognitive functioning since SCN3B and HOPX were lower expressed and DSP was higher expressed in hippocampal region of patients with Alzheimer’s disease [2]. This suggests that these genes may have a function in shear stress and/or hypoxia related cognitive functioning.

### A previously unknown expression profile of DSP, HOPX and SCN3B in intracranial ECs

**Fig. 4:**
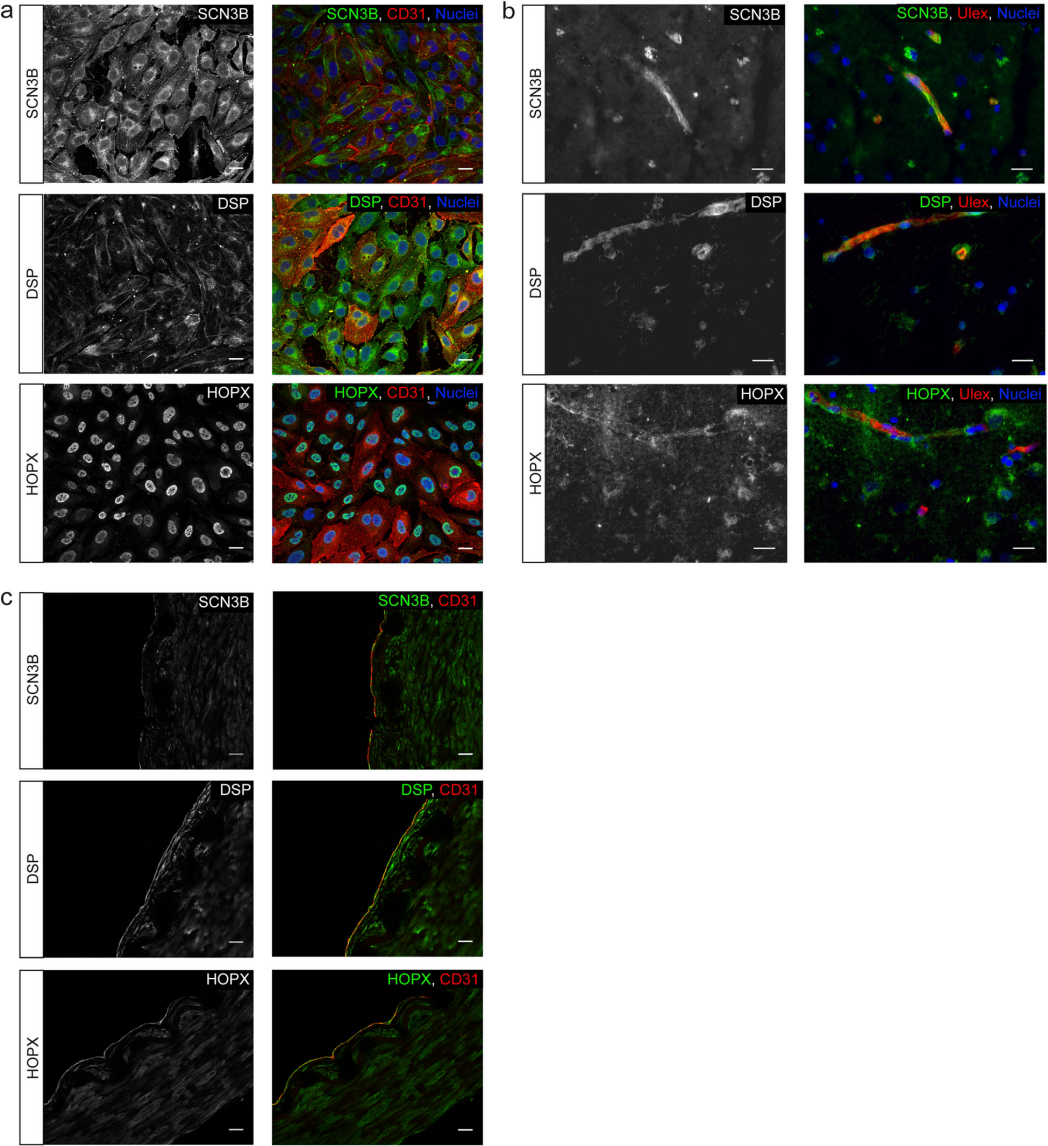
Expression profile of SCN3B, DSP and HOPX in brain ECs. Localization of DSP, HOPX and SCN3B in (**a**) human brain ECs in vitro*,* (**b)** post-mortem human cortical microvasculature and (**c**) post-mortem human BA (of one representative patient). SCN3B upper panel, DSP middle panel and HOPX lower panel. Overlay depicts SCN3B, DSP or HOPX in green, ECs in red (CD31 for **a** and **c**, Ulex for **b**) and (**a**, **b**) nuclei in blue. **a**, **b**, **c** Scale bar represents 20 μm

To investigate the presence of these genes in intracranial ECs in more detail, we studied the protein expression of SCN3B, DSP and HOPX in vitro and in post-mortem brain tissue. Immunocytochemistry of SCN3B, DSP and HOPX in intracranial ECs in vitro showed a perinuclear localization of SCN3B, while DSP and HOPX showed both nuclear as well as perinuclear localization (Fig. 4a). In addition, we further validated the expression of these proteins in post-mortem human brain microvasculature (Fig. 4b) and in the BA of the patients used for RNA sequencing (Fig. 4c and data not shown). Although the expression is not specific for ECs, all three genes are expressed in ECs of the brain. The expression in ECs underscores again the role of these three genes in human brain EC function.

## Discussion

In this study we performed RNA sequencing on human ECs of paired macroscopically normal carotid and basilar arteries. We predominantly detected differential expression of genes involved in immunoquiescence and response to EC damage. Moreover, we discovered the differential expression of genes related to perfusion and cognition in particular SCN3B, HOPX and DSP. Consistently, we show that SCN3B, HOPX and DSP are sensitive to hypoxia and/or shear stress in vitro, suggesting a novel role of these genes in the susceptibility of intracranial ECs to hypoxia and aberrant shear stress, processes involved in vascular cognitive functioning.

In this paper we strengthened the immunoquiescent and revealed a unique damage response phenotype of the intracranial artery ECs, by showing a decreased expression of immune-responsive genes, and different regulation of EC damage-related genes in the intracranial ECs compared to the extracranial ECs. The involvement of the intracranial artery ECs in immunoquiescence and EC damage has not extensively been studied, however cell based assays showed a decrease in immune responsiveness in brain ECs compared to peripheral ECs [59]. Furthermore, it has been reported that human intracranial arteries display a higher anti-oxidant activity compared to extracranial arteries [9]. Besides this limited amount of literature on the intracranial arteries, an extensive amount of research is performed on the intracranial microvasculature. Intracranial ECs of the microvasculature of the brain form a tight barrier between the blood and the underlying brain tissue, known as the blood-brain-barrier. ECs of the brain microvasculature regulate permeability and can maintain an immunoquiescent state. Besides that, cell adhesion, differentiation, proliferation and response to oxidative stress and inflammation are reduced in the ECs of the blood-brain barrier, thereby protecting the brain tissue. This is in accordance with the EC damage phenotype of the BA ECs, which we reported here. However, in our dataset, specific blood-brain-barrier related genes, like ABC-transporters and tight junction proteins, were not differentially expressed in the BA and CCA, except for ABCB1 and claudin 5 and 10 which were higher expressed in the intracranial artery ECs compared to the extracranial artery ECs. This suggests different expression profiles of the intracranial artery ECs compared to the ECs of the brain microvasculature.

In our data set we revealed the expression of a number of genes yet unknown to be present in intracranial arterial ECs. We found that these genes are not only expressed in arterial ECs but also differentially expressed between the intracranial- and the extracranial arterial ECs. Our data are the first human expression profiling studies of these arteries. Of the 900 differentially expressed genes, we identified 15 genes reported to be involved in both perfusion and cognition. Analyzing these genes upon hypoxia and/or shear stress conditions, resulted in a set of three genes that are differentially expressed in the intracranial ECs, previously linked to cognition and, in the current study, found to play a role in endothelial susceptibility to hypoxia and/or shear stress. One of the key genes we found to be highly expressed in the intracranial ECs compared to the extracranial ECs is DSP. In general, DSP is known to be a major component of desmosomes that facilitate adhesion in epithelial cells, although to date desmosomes have not been described in endothelial cells. On the other hand, DSP was reported to be a component of the complex adherence junction, *Complexus adhaerentes,* which is present in specific endothelial cells like lymphatic, umbilical vein and lung microvascular endothelial cells [29, 51, 58]. This complex adherence junction consists of E-cadherin, catenins and DSP and is molecularly and structurally different from desmosomes and adherence junctions. Interestingly, loss of DSP causes a weakening of endothelial cell-cell contacts [15]. Although the function of DSP in intracranial ECs has not been investigated, its higher expression suggests stronger cell-cell contact between intracranial ECs compared to extracranial ECs, as is also seen in the blood-brain-barrier of the cerebral microvasculature. Decreased expression of DSP upon shear stress in vitro in our endothelial cell cultures suggests a loss of the complex adherence junction upon shear stress. In literature, shear stress results in a reorganization of adherence junctions facilitating the alignment of the ECs [37, 61], which suggests that the complex adherence junctions and/or DSP may also be involved in this reorganization.

Another gene that we found to be highly expressed in the intracranial arterial ECs compared to the extracranial arterial ECs is the transcription factor HOPX. Endothelial expression of HOPX was only reported before in the cerebral cortex [53]. HOPX is known to be expressed in cardiac progenitor cells that will later develop into cardiomyoblasts [25]. Furthermore, HOPX was found to regulate primitive hematopoiesis, however loss of HOPX does not affect endothelial fate specification [39]. Interestingly, HOPX is higher expressed in intracranial ECs compared to extracranial ECs. In more detail, our data showed that HOPX is induced under hypoxic conditions, therefore we suggest a role of HOPX in hypoxic conditions in the intracranial ECs. Since HOPX is a transcription factor, it may be involved in establishing or maintaining the unique intracranial signature upon hypoxic conditions.

Lastly, in our dataset we found SCN3B as a gene differentially expressed in arterial ECs. We detected a decreased expression of SCN3B in intracranial arterial ECs compared to the extracranial arterial ECs (in comparison with DSP and HOPX which were more highly expressed in the intracranial ECs). SCN3B is known to inactivate sodium channels and regulate the action potentials in neurons and myocytes. Sodium channels are mainly present in epithelial cells, however, recent data showed also expression of these channels in ECs [27]. In general, ECs are protected by a glycocalyx that buffers sodium entry into the cell and ECs display a low expression of sodium channels. A damaged glycocalyx and an increase in the expression of sodium channels may facilitate Na + entry into the ECs, thereby triggering enhanced endothelial permeability [62]. In our data set, we found a lower expression of SCN3B in the intracranial ECs compared to the extracranial ECs, which suggests a different Na + entry regulation in the intracranial ECs. The decrease of SCN3B upon high shear stress suggests an activation of the sodium channels and an increase in permeability. In addition, the increase of SCN3B in endothelial cells upon hypoxia suggests an inactivation of the sodium channels and thereby a decrease in permeability, which has been documented before for hypoxia [38]. The proposed involvement of these three genes in adhesion and permeability suggests a protective role of the intracranial artery ECs against damage induced by perfusion changes (hypoxia and/or shear stress).

Next to the involvement of these three genes in perfusion, a previous study showed that in the hippocampus of patients with Alzheimer’s disease, SCN3B and HOPX are downregulated and DSP is upregulated (sections of left hippocampus, total tissue) [2], suggesting their involvement in cognition. However, the vasculature was not addressed here. In our preliminary data, we could verify this trend in mRNA expression in the brain capillaries of an Alzheimer’s disease mouse model (APP/PS1 transgenic mouse model). SCN3B and HOPX seem to be lower expressed, and DSP higher expressed in the brain capillaries of these mice compared to wild type mice (not significant, data not shown). As we postulate here that DSP, SCN3B and HOPX are genes involved in the susceptibility of intracranial ECs against perfusion changes and since we know that they are involved in Alzheimer’s disease, further research should focus on the expression of these genes in intracranial ECs of (early and late stage) patients with vascular cognitive disorders. Currently, biomarkers and therapeutic approaches for VCI are missing, due to a lack of understanding of the molecular regulation of VCI and more specific the EC function in VCI. Since the ECs are key in transferring signals from the blood to the tissue and especially in sensing perfusion changes, we suggest EC molecular targets as key in therapeutic intervention approaches for VCI [21].

Our transcriptome analysis on intracranial artery ECs is based on unique post-mortem material and all analyses were performed per individual rather than in vivo or in vitro models. All published transcriptomic studies on intracranial ECs are restricted to the microvasculature of the brain, and data on the transcriptome of the intracranial artery ECs are lacking, especially in humans. Most studies on the transcriptional regulation of intracranial ECs are performed in mice and focus on the brain microvasculature [15]. The comparison of gene expression in brain microvasculature ECs with ECs from the liver, lung and kidney in mice, revealed a distinct molecular architecture of these EC populations in that only intracranial ECs in the embryonic stage exhibit canonical Wnt signaling [48]. Moreover, seven different vascular beds in the mouse, among which the brain, were used in RNA sequencing, to unravel the transcriptional regulation during development and brain differentiation [24]. Furthermore, single-cell RNA sequencing analysis revealed vascular and vessel-associated cell types in mouse brain and lung, creating a comprehensive molecular dataset for future cell specific research [20]. Recently, the gene expression profile of mural cells, astrocytes, oligodendrocyte precursors, microglia, fibroblast-like cells and the brain endothelial cells along the arteriovenous axis in mice was reported, revealing a specific molecular blueprint of the ECs from an arterial to venous origin [60]. In human, isolation of all types of brain cells, including endothelial cells, was performed followed by culturing of these cells for 3 weeks after which single-cell deep sequencing was performed. Gene expression profiles of human astrocytes, microglia, neurons, oligodendrocytes and endothelial cells were determined and differences between murine and human expression profiles were established [8, 52, 66]. In these datasets, both DSP and SCN3B were reported to be expressed in the mouse intracranial ECs, specifically in arterial ECs, whereas HOPX was not detected [20, 24, 60]. However, the comparison of gene expression profiles in human intracranial artery ECs and extracranial ECs and the function of these genes in arterial intracranial ECs was not investigated before.

In our study Amyotrophic Lateral Sclerosis (ALS) patients are overrepresented, since in our institution brain autopsies are more often performed on ALS patients, than on individuals with a non-neurological cause of death. A limitation of our study is that ALS is a genetic driven disease, which may influence our gene analysis [1]. However, we could verify that ALS patients did not have a different expression profile for our genes of interest compared to the non-ALS patients (Additional file 1: Figure S2), although the number of patients for this analysis was low (6 vs. 3 patients). To study the transcriptional profile of the ECs of ALS patients vs. non-neurological controls, an extensive study with more patients and their controls needs to be performed.

The post-mortem nature of the samples used in our study could raise the question if PMD can explain the gene expression differences found in our analysis [13]. The mRIN values of the samples showed no significant RNA degradation (Additional file 1: Table S1). Furthermore, we found no correlation between PMD and mRIN values of the individuals used in our analysis (Additional file 1: Figure S3). Also here, the number of individuals for this correlation is low (11 patients). More individuals should be included for an extensive analysis between PMD and mRIN values. However, the gene expression differences analyzed in our manuscript were performed per individual. Therefore the effect of PMD on gene expression is assumed to be equal between the intracranial and extracranial endothelial cells analyzed. Furthermore, it is suggested that certain tissues, including the brain, have little sensitivity to post-mortem mRNA degradation [67].

With our data we found evidence for a protective phenotype of the human intracranial artery ECs against EC damage thereby they may play a role in vascular cognitive functioning. We furthermore identified three genes, DSP, SCN3B and HOPX, with a previously unknown function in intracranial artery ECs, that are susceptible to hypoxia and/or shear stress. Our data showed that we are able to collect post-mortem ECs with laser capture microdissection that can be used in further functional studies, here in relation to perfusion and cognition, but all studies involved in intracranial ECs can greatly benefit from these data.

## Conclusions

In conclusion, the present profiling study indicates for the first time that human intracranial arterial endothelial cells, compared to extracranial arterial endothelial cells, display a protective phenotype against endothelial damage. Our study highlights the importance of such genes in future studies into their role in the occurence of vascular cognitive impairment.

## Supplementary information

**Additional file 1:** **Table S1.** mRIN value with z-score and *P*-value for each sample Two samples per patient, first sample derived from BA, second sample derived from CCA. Samples with P-value > 0.05 were used in RNA sequencing analysis [1]. **Table S2.** List of primers used in this study Full names with gene symbols and primer sequences used for qPCR analysis. **Table S3.** Differential expression of genes per gene set analyzed. (A) Gene set consisting of genes upregulated in primary endothelial cell types (lung, aortic, iliac, dermal, and colon) treated with interferon-gamma (IFNγ) (MSigDB ID: M4551). Almost all genes induced by IFNγ were higher expressed in extracranial ECs compared to intracranial ECs. (B) Genes differentially expressed in intracranial ECs and extracranial ECs and involved in EC damage response. Subcategorized for response to oxidative stress, EC proliferation, inflammatory response, cell adhesion, EC differentiation and vascular permeability. (C) 15 genes differentially expressed in intracranial ECs and extracranial ECs and related to perfusion and cognition. LogFC < 0 is expression elevated in intracranial ECs, logFC > 0 is elevated in extracranial ECs. **Figure S1.** Expression stability of reference targets vWF and Pecam1/CD31 are most stable (lowest geNorm M, with M as gene stability value [2]) between BA and CCA samples (*n* = 11 patients). (1) and (2) refers to different primer pairs. CD34 = cluster of differentiation 34, Cdh5 = cadherin 5, Kdr = Kinase Insert Domain Receptor/Vascular Endothelial Growth Factor Receptor 2, Pecam = Platelet And Endothelial Cell Adhesion Molecule 1/CD31, Rplp0 = Ribosomal Protein Lateral Stalk Subunit P0, vWF = Von Willebrand Factor. **Figure S2.** Normalized expression of DSP, HOPX and SCN3B in ALS versus non- ALS patients. Beeswarm plots with the normalized expression (log2CPM) of (a) DSP, (b) HOPX and (c) SCN3B in the BA and CCA samples used for RNA sequencing. No differences between ALS and non-ALS patient are detected. **Figure S3.** Correlation between PMD and mRIN values. PMD of (a) BA samples and (b) CCA samples (*n* = 11 individuals) correlated to their mRIN values. No significant correlation found for BA and CCA samples. Dots are individuals used in our analysis (see also Table 1 for PMD perindividual).

## Data Availability

The datasets used and analyzed during the current study is available from the corresponding author on request and will be available in the European Genome-Phenome Archive (EGA) upon publication.
