# Supplementary material for: Profiling the unique protective properties of intracranial arterial endothelial cells
Source: Acta Neuropathol Commun. 2019 Oct 14;7:151. doi: 10.1186/s40478-019-0805-4 (PMC6792251; doi:10.1186/s40478-019-0805-4)
Supplement: Supplementary file 1 — Additional file 1: Table S1. mRIN value with z-score and P-value for each sample Two samples per patient, first sample derived from BA, second sample derived from CCA. Samples with P-value > 0.05 were used in RNA sequencing analysis [1]. Table S2. List of primers used in this study Full names with gene symbols and primer sequences used for qPCR analysis. Table S3. Differential expression of genes per gene set analyzed. (A) Gene set consisting of genes upregulated in primary endothelial cell types (lung, aortic, iliac, dermal, and colon) treated with interferon-gamma (IFNγ) (MSigDB ID: M4551). Almost all genes induced by IFNγ were higher expressed in extracranial ECs compared to intracranial ECs. (B) Genes differentially expressed in intracranial ECs and extracranial ECs and involved in EC damage response. Subcategorized for response to oxidative stress, EC proliferation, inflammatory response, cell adhesion, EC differentiation and vascular permeability. (C) 15 genes differentially expressed in intracranial ECs and extracranial ECs and related to perfusion and cognition. LogFC < 0 is expression elevated in intracranial ECs, logFC > 0 is elevated in extracranial ECs. Figure S1. Expression stability of reference targets vWF and Pecam1/CD31 are most stable (lowest geNorm M, with M as gene stability value [2]) between BA and CCA samples (n = 11 patients). (1) and (2) refers to different primer pairs. CD34 = cluster of differentiation 34, Cdh5 = cadherin 5, Kdr = Kinase Insert Domain Receptor/Vascular Endothelial Growth Factor Receptor 2, Pecam = Platelet And Endothelial Cell Adhesion Molecule 1/CD31, Rplp0 = Ribosomal Protein Lateral Stalk Subunit P0, vWF = Von Willebrand Factor. Figure S2. Normalized expression of DSP, HOPX and SCN3B in ALS versus non- ALS patients. Beeswarm plots with the normalized expression (log2CPM) of (a) DSP, (b) HOPX and (c) SCN3B in the BA and CCA samples used for RNA sequencing. No differences between ALS and non-ALS patient are detected. Figu [file 40478_2019_805_MOESM1_ESM.pdf]

Additional file 1

Additional file 1: Table and Figure legends

Table S1 mRIN value with z-score and P-value for each sample Two samples per patient, first sample derived from BA, second sample derived from CCA. Samples with P-value > 0.05 were used in RNA sequencing analysis [1].

Table S2 List of primers used in this study Full names with gene symbols and primer sequences used for qPCR analysis.

Table S3 Differential expression of genes per gene set analyzed. (A) Gene set consisting of genes upregulated in primary endothelial cell types (lung, aortic, iliac, dermal, and colon) treated with interferon-gamma (IFNγ) (MSigDB ID: M4551). Almost all genes induced by IFNγ were higher expressed in extracranial ECs compared to intracranial ECs. (B) Genes differentially expressed in intracranial ECs and extracranial ECs and involved in EC damage response. Subcategorized for response to oxidative stress, EC proliferation, inflammatory response, cell adhesion, EC differentiation and vascular permeability. (C) 15 genes differentially expressed in intracranial ECs and extracranial ECs and related to perfusion and cognition. LogFC < 0 is expression elevated in intracranial ECs, logFC > 0 is elevated in extracranial ECs.

Figure S1 Expression stability of reference targets vWF and Pecam1/CD31 are most stable (lowest geNorm M, with M as gene stability value [2]) between BA and CCA samples (n=11 patients). (1) and (2) refers to different primer pairs. CD34 = cluster of differentiation 34, Cdh5 = cadherin 5, Kdr = Kinase Insert Domain Receptor/Vascular Endothelial Growth Factor Receptor 2, Pecam = Platelet And Endothelial Cell Adhesion Molecule 1/CD31, Rplp0 = Ribosomal Protein Lateral Stalk Subunit P0, vWF = Von Willebrand Factor.

Figure S2 Normalized expression of DSP, HOPX and SCN3B in ALS versus non-ALS patients. Beeswarm plots with the normalized expression (log2CPM) of (a) DSP, (b) HOPX and (c) SCN3B in the BA and CCA samples used for RNA sequencing. No differences between ALS and non-ALS patient are detected.

Figure S3 Correlation between PMD and mRIN values. PMD of (a) BA samples and (b) CCA samples (n=11 individuals) correlated to their mRIN values. No significant correlation found for BA and CCA samples. Dots are individuals used in our analysis (see also table 1 for PMD per individual).

References

1. Feng H, Zhang X, Zhang C (2015) mRIN for direct assessment of genome-wide and gene-specific mRNA integrity from large-scale RNA-sequencing data. Nat Commun 6:7816. doi:10.1038/ncomms8816
2. Hellemans J, Mortier G, De Paepe A, Speleman F, Vandesompele J (2007) qBase relative quantification framework and software for management and automated analysis of real-time quantitative PCR data. Genome Biol 8:R19. doi:10.1186/gb-2007-8-2-r19

Table S1

Patient	Sample	mRIN	z-score	P-value
1	s001	0.001	-0.179	0.429
	s002	0.013	0.719	0.764
2	s003	-0.008	-0.870	0.192
	s004	0.027	1.738	0.959
3	s005	0.009	0.254	0.600
	s006	0.024	1.555	0.940
4	s007	0.009	0.423	0.664
	s008	-0.012	-1.116	0.132
5	s009	0.009	0.449	0.673
	s010	-0.005	-0.655	0.256
6	s011	0.022	1.355	0.912
	s012	0.007	0.278	0.609
7	s013	0.001	-0.146	0.442
	s014	-0.001	-0.358	0.360
8	s015	0.002	-0.076	0.470
	s016	0.017	1.004	0.842
9	s017	-0.008	-0.870	0.192
	s018	-0.014	-1.291	0.098

Table S2

Full name (provided by HGNC)	Symbol	Sequence forward primer	Sequence reverse primer
Actin alpha 2, smooth muscle	ACTA2	CCAGCAGATGTGGATCAGCAA	TGCTAGAGACAGAGAGGAGCA
Beta-2-microglobulin	B2M	TTTCATCCATCCGACATTG	CGGCAGGCATACTCATCTTT
Cannabinoid receptor 1	CNR1	TAACAGCATGGGGGACTCGG	CTCACAGAGCCTCGGCAGAC
Collagen type I alpha 2 chain	Col1A2	TTTGGACATCGGTGGTGCT	TGCCCACAATTTAAGCAAGTAGA
Collagen type III alpha 1 chain	Col3A1	CGTTGGCCCTGTTTGCTTTT	TTGTCGGTCACTTGCACTGG
Desmoplakin	DSP	GTGTCGTCCAAGGGCTTACC	TAGCAACCACTCCCAACTGAC
HOP homeobox	HOPX	AACAAGGTCGACAAGCACCC	GCTGTCAATGCCTGCCATCT
Kruppel like factor 2	KLF2	AGTGGCATCTTCTCTCCCACC	GGCCCTTATTTCTCACAAGGC
Myosin light chain 4	MYL4	AGCACATCATGTCAGGGTGA	TGGCCGTGTTAGAGCTGTTT
Neuregulin 1	NRG1	ACCCCTGATTCCTACCGAGA	AGCTTGGCGTGTGGAAATCT
Nitric oxide synthase 3	NOS3	CTCATGGGCACGGTGATG	ACCACGTCATACTCATCCATACAC
Phosphodiesterase 4D	PDE4D	AGAGGAGGCAGTAGGGGAAG	CCTGGCAGATGACAGTGAGG
Platelet and endothelial cell adhesion molecule 1	PECAM1	ATTCCTGAAGTCCGGATCTATG	ACCTTGGATGGCCTCTTTCT
Ryanodine receptor 3	RYR3	AAAAGAAGAAGCGGCGGTGT	GCGAAGGCTACAAACAGAGCA
S100 calcium binding protein A4	S100A4	CTGCATCGCCATGATGTGTA	CCCAACCACATCAGAGGAGT
Sodium voltage-gated channel beta subunit 3	SCN3B	ACCTTGCCATCCCATCTGAG	GTCCAGTCCCTCAGGTGTTC
Thrombomodulin	THBD	GCATTACAGCTGGAGAAGACCC	TTCTCCTCCCTCTAATCACCCC
Tight junction protein 1	TJP1	AAGTCACACTGGTGAAATCC	CTCTTGCTGCCAAACTATCT
Vascular endothelial growth factor A	VEGFA	CATCTTCAAGCCATCCTGCTGTGTG	GCATGGTGATGTTGGACTCCT
Vimentin	VIM	TCCCTCTGGTTGATACCCACT	CGTGATGCTGAGAAGTTTCGT
Von Willebrand factor	vWF	TGCTGACACCAGAAAAGTGC	CACAGGAGCAGGTGTCGTAA

Table S3A

Response to IFNγ

hgnc_symbol	entrezgene	LogFC	-Log10(pVal)
CX3CL1	6376	-0.933	3.701
RABL3	285282	-0.437	0.627
IFIH1	64135	-0.316	0.465
DTX3L	151636	-0.209	0.251
RARRES3	5920	-0.117	0.043
VAMP5	10791	-0.082	0.055
SEPT4	5414	0.088	0.074
SLC25A28	81894	0.128	0.195
TNFSF10	8743	0.152	0.047
WARS	7453	0.172	0.309
IL18BP	10068	0.211	0.152
UBE2L6	9246	0.318	0.249
LAP3	51056	0.335	0.399
TRIM22	10346	0.337	0.593
CASP1	834	0.346	0.149
LGALS9	3965	0.351	0.127
PARP9	83666	0.361	0.418
RNF213	57674	0.380	1.714
GOLM1	51280	0.385	0.714
NLRC5	84166	0.472	0.553
MX1	4599	0.472	0.497
IFI44L	10964	0.485	0.508
DDX60	55601	0.543	0.973
APOL2	23780	0.608	0.711
PPP3CA	5530	0.634	0.950
HLA-A	3105	0.636	1.440
SSPN	8082	0.686	1.712
HLA-C	3107	0.700	1.846
HSD17B11	51170	0.703	0.923
HLA-E	3133	0.773	1.986
GBP1	2633	0.781	1.587
MLKL	197259	0.787	0.637
HLA-DRB1	3123	0.799	0.535
SERPING1	710	0.827	4.819
APOL1	8542	0.851	1.027
HAUS7	55559	0.851	0.666
BATF2	116071	0.854	0.408
SAMD9L	219285	0.887	2.628
C1S	716	0.893	1.601
LGALS3BP	3959	0.940	1.179
HLA-B	3106	0.950	2.277
GBP3	2635	1.020	1.795
GBP4	115361	1.070	1.308
PARP14	54625	1.122	4.088
GIMAP7	168537	1.148	0.598

APOL3	80833	1.163	2.085
HLA-DPA1	3113	1.168	0.645
HLA-DRB5	3127	1.201	0.908
IFI35	3430	1.364	1.015
CD274	29126	1.416	1.066
BST2	684	1.540	1.077
HLA-DMA	3108	1.720	1.944
OAS2	4939	1.754	1.383
CFH	3075	1.819	2.641
CEACAM1	634	1.842	1.777
ST8SIA4	7903	2.076	2.158
HLA-DQB1	3119	2.081	1.407
HLA-DQA1	3117	2.131	1.890
APOL4	80832	2.205	2.083
PLA1A	51365	2.302	1.339
OAS1	4938	2.487	1.783
SAMHD1	25939	3.100	5.504
CXCL9	4283	3.228	2.468

Table S3B

ECs damage response

Response to oxidative stress

hgnc_symbol	entrezgene	LogFC	-Log10(pVal)
NOS1	4842	-4.312	4.619
STC2	8614	-4.121	4.254
DHCR24	1718	-2.846	2.446
SLC7A11	23657	-2.710	2.621
RBM11	54033	-2.374	1.351
CYCS	54205	-2.045	4.846
PDGFD	80310	3.757	8.124
SELENOP	6414	3.617	4.353
CD38	952	3.061	3.258
TRPM2	7226	2.680	1.709
APOE	348	2.411	1.645
ALDH3B1	221	2.189	1.863
PDGFRA	5156	2.037	3.421

EC proliferation

hgnc_symbol	entrezgene	LogFC	-Log10(pVal)
FLT4	2324	-4.995	5.260
DLL4	54567	-3.389	2.249
THBS1	7057	-2.361	4.219
ALDH1A2	8854	4.036	4.386
APOE	348	2.411	1.645
BMP2	650	2.124	2.524
HTR2B	3357	2.068	2.912
CDH13	1012	2.372	6.421

Inflammatory response

hgnc_symbol	entrezgene	LogFC	-Log10(pVal)
KRT16	3868	-6.920	8.020
SCN9A	6335	-5.197	8.864
P2RX1	5023	-5.159	8.587
THBS1	7057	-2.361	4.219
CDO1	1036	4.222	5.112
CD163	9332	3.871	3.476
CHST2	9435	3.729	4.120
TNFRSF21	27242	3.690	3.960
TNFSF4	7292	3.430	3.555
SIGLEC1	6614	3.429	3.664
KIT	3815	3.304	3.018
CXCL9	4283	3.228	2.469
BMPR1B	658	3.188	5.289
CCL4	6351	2.799	3.382
CD180	4064	2.699	1.973
NAIP	4671	2.679	2.321
C3AR1	719	2.436	3.626
CLEC7A	64581	2.429	1.707
TLR1	7096	2.424	2.154
SYK	6850	2.359	1.905
CXCR4	7852	2.322	1.936
LXN	56925	2.301	1.748
AOAH	313	2.295	2.218
PRKCQ	5588	2.233	1.729
TLR4	7099	2.126	4.189
BMP2	650	2.124	2.524
GBP5	115362	2.118	1.792
ZAP70	7535	2.078	1.346

Cell adhesion

hgnc_symbol	entrezgene	LogFC	-Log10(pVal)
CD24	100133941	-4.784	5.267
EPHA7	2045	-4.118	3.827
LEF1	51176	-3.176	4.031
JAG2	3714	-3.000	2.552
TGFBI	7045	-2.431	7.127
THBS1	7057	-2.361	4.219
FZD7	8324	-2.118	3.574
NRG1	3084	6.365	8.561
SFRP1	6422	5.989	7.465
EPHA3	2042	5.101	8.700
CD209	30835	4.691	5.125
LAMA2	3908	4.421	9.904
CD28	940	4.036	3.062
PIK3R6	146850	3.763	2.983
TNFRSF21	27242	3.690	3.960
PDE3B	5140	3.614	5.697

TNFSF4	7292	3.430	3.555
RASAL3	64926	3.193	3.569
VAV3	10451	2.875	2.867
PTPRZ1	5803	2.875	1.865
KIF26B	55083	2.760	3.502
IL7R	3575	2.693	3.431
ITGA4	3676	2.634	2.023
FMN1	342184	2.614	3.703
PLET1	349633	2.597	2.185
HAS2	3037	2.542	1.905
CD80	941	2.506	1.404
LILRB2	10288	2.463	1.755
FERMT3	83706	2.443	1.675
SYK	6850	2.359	1.905
CD247	919	2.312	1.762
CD3E	916	2.284	1.876
HLA-DMB	3109	2.262	1.700
PRKCQ	5588	2.233	1.729
HLA-DQA1	3117	2.131	1.890
BMP2	650	2.124	2.524
DPP4	1803	2.117	1.357
PTPRC	5788	2.097	3.130
ZAP70	7535	2.078	1.346
HLA-DQB1	3119	2.076	2.158
RAC2	5880	2.061	1.324
CDH13	1012	2.037	6.421

EC differentation

hgnc_symbol	entrezgene	LogFC	-Log10(pVal)
HEY1	23462	-4.932	5.868
PDE4D	5144	-2.943	9.053
NRG1	3084	6.365	8.561
BMPER	168667	2.318	1.381

Vascular permeability

hgnc_symbol	entrezgene	LogFC	-Log10(pVal)
PTP4A3	11156	-2.843	4.916
NPR1	4881	2.510	4.823
SLIT2	9353	2.299	5.967

Table S3C

Perfusion and cognition related genes

hgnc_symbol	entrezgene	LogFC	-Log10(pVal)
CACNA1S	779	-7.512	10.333
STRA6	64220	-4.616	3.695
NOS1	4842	-4.312	4.619
DSP	1832	-4.049	3.613

HRH2	3274	-4.035	1.895
HOPX	84525	-3.787	3.343
PDE4D	5144	-2.943	9.053
GABRB3	2562	-2.275	2.004
NRG1	3084	6.365	8.561
NRXN2	9379	4.006	3.994
MYL4	4635	3.778	5.711
CNR1	1268	2.890	3.065
RYR3	6263	2.818	9.264
SCN3B	55800	2.402	1.305
MUSK	4593	2.201	1.946

Figure S1

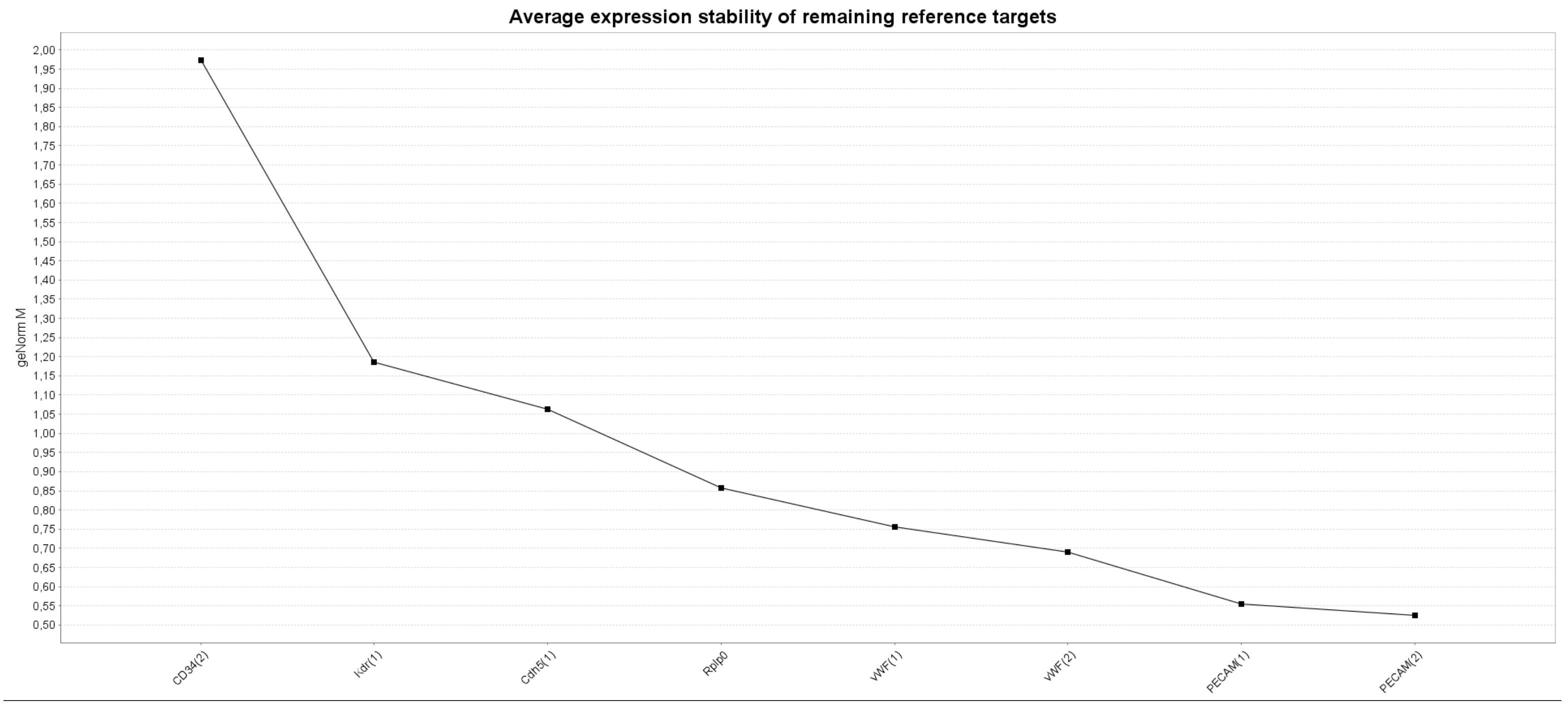
Average expression stability of remaining reference targets
geNorm M
2,00
1,95
1,90
1,85
1,80
1,75
1,70
1,65
1,60
1,55
1,50
1,45
1,40
1,35
1,30
1,25
1,20
1,15
1,10
1,05
1,00
0,95
0,90
0,85
0,80
0,75
0,70
0,65
0,60
0,55
0,50
CD34(2)
Kdr(1)
Cdh5(1)
Rplp0
vWF(1)
vWF(2)
PECAM(1)
PECAM(2)

Figure S2

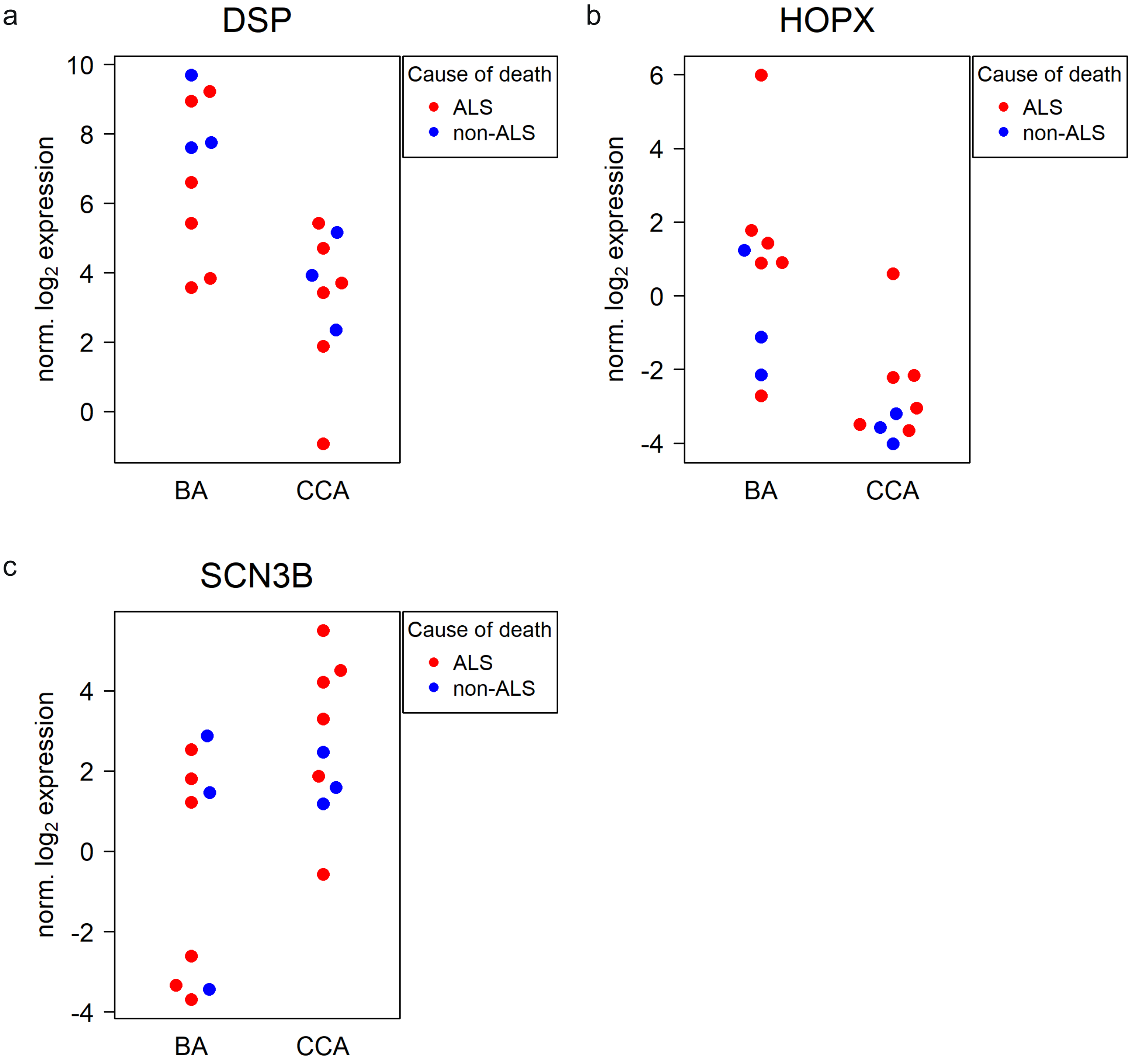
a
DSP
Cause of death
ALS
non-ALS
norm. log2 expression
10
8
6
4
2
0
BA
CCA
b
HOPX
Cause of death
ALS
non-ALS
norm. log2 expression
6
4
2
0
-2
-4
BA
CCA
c
SCN3B
Cause of death
ALS
non-ALS
norm. log2 expression
4
2
0
-2
-4
BA
CCA

Figure S3

a

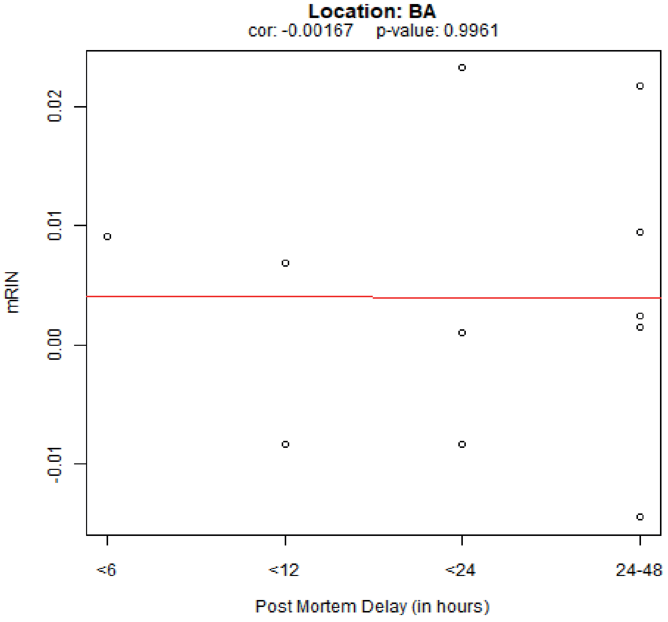
Location: BA
cor: -0.00167 p-value: 0.9961
mRIN
0.02
0.01
0.00
-0.01
<6
<12
<24
24-48
Post Mortem Delay (in hours)

b

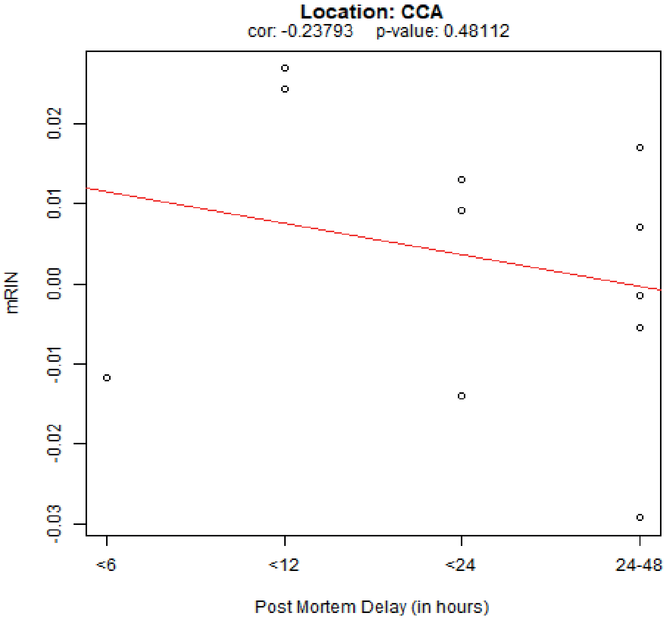
Location: CCA
cor: -0.23793 p-value: 0.48112
mRIN
0.02
0.01
0.00
-0.01
-0.02
-0.03
<6
<12
<24
24-48
Post Mortem Delay (in hours)
